# Symptoms and Signs Associated with Postpartum Thyroiditis

**DOI:** 10.1155/2014/531969

**Published:** 2014-10-27

**Authors:** Maureen Groer, Cecilia Jevitt

**Affiliations:** ^1^University of South Florida College of Nursing, 12910 Bruce B. Downs Boulevard, Tampa, FL 33612, USA; ^2^Yale University School of Nursing, P.O. Box 27399, West Haven, CT 06515-7399, USA

## Abstract

*Background.* Postpartum thyroiditis (PPT) is a common triphasic autoimmune disease in women with thyroid peroxidase (TPO) autoantibodies. This study evaluated women's thyroid disease symptoms, physical findings, stress levels, and thyroid stimulating hormone (TSH) levels across six postpartum months in three groups, TPO negative, TPO positive, and PPT positive women. *Methods.* Women were recruited in midpregnancy (*n* = 631) and TPO status was determined which then was used to form the three postpartum groups. The three groups were compared on TSH levels, thyroid symptoms, weight, blood pressure, heart rate, a thyroid exam, and stress scores. *Results.* Fifty-six percent of the TPO positive women developed PPT. Hypothyroid group (*F* (2, 742) = 5.8, *P* = .003) and hyperthyroid group (*F* (2, 747) = 6.6, *P* = .001) subscale scores differed by group. Several symptoms and stress scores were highest in the PPT group. *Conclusions.* The normal postpartum is associated with many symptoms that mimic thyroid disease symptoms, but severity is greater in women with either TPO or PPT positivity. While the most severe symptoms were generally seen in PPT positive women, even TPO positive women seem to have higher risk for these signs and symptoms.

## 1. Introduction

The postpartum is considered a vulnerable time for the development or recurrence of some autoimmune diseases (ADs), particularly those caused by excessive T helper 1 (Th1) lymphocyte mediated processes (eg. rheumatoid arthritis (RA)), multiple sclerosis (MS), and postpartum thyroiditis (PPT). These ADs often ameliorate during pregnancy but then exacerbate or occur for the first time in the postpartum period [1]. This is thought to be due to the return of Th1 mediated immunity, which is suppressed in pregnancy [2]. The most common postpartum autoimmune disease (AD) is postpartum thyroiditis (PPT), a thyroid dysfunction that occurs within the first year after delivery or miscarriage [3]. PPT occurs during the early postpartum in approximately half of women who have autoantibodies to the enzyme, thyroid peroxidase, during the first trimester of pregnancy [4]. Thyroid peroxidase is a microsomal enzyme that is required in the synthesis of thyroid hormone, but many women who have this autoantibody remain euthyroid. PPT usually has a triphasic course, from euthyroidism, to a short hyperthyroid phase (22%) followed by a longer period of hypothyroidism (48%) and a recovery to euthyroidism [5]. Variants of this course occur. Recently it has been shown that up to fifty percent of women with PPT remain hypothyroid at one year [6]. There is significant risk for women with PPT (up to 50%) to develop permanent thyroid disease over time [3, 6]. The pathophysiology involves Th1 cells, autoantibodies, and autoreactive T helper 2 (Th2) cells that promote antithyroid autoantibody production. Cytotoxic T cells and NK cells also participate in a direct destruction of the gland [7]. TPO autoantibodies are able to bind to thyrocytes and activate complement, which sets in motion antibody dependent cytotoxic mechanisms which involve further destruction of the thyrocytes.

Symptoms that women with PPT experience are thought to be related to the phase of disease. PPT symptoms during the hyperthyroid phase are usually short-lived and in the hypothyroid phase are particularly likely to be underdiagnosed, as many women consider their experiences as “normal” postpartum symptoms. Routine TPO antibody screening is not done in pregnancy and many TPO positive women have no or subclinical symptoms of thyroid dysfunction, so risk for PPT cannot be predicted for the individual untested woman unless she has had a previous episode of the disease.

We examined differences across the course of the postpartum in TPO positive women compared to a TPO negative comparison group. A list of common symptoms of hypo- and hyperthyroidism was constructed, and women reported on the occurrence and severity of each symptom at 7 consecutive postpartum assessments. The current report compares symptoms, TSH levels, other hormones, and physical findings in three groups, TPO negative, TPO positive, and PPT positive women across six postpartum months.

## 2. Method

Institutional Review Board (IRB) approval was achieved and informed consent gathered at the initiation of the study. Pregnant women (*n* = 631) were recruited at prenatal clinics. Study participants were women first measured for the TPO autoantibody after the first trimester and before 25 weeks of gestation and identified as either TPO positive or negative at that time. Exclusion criteria included the following: age less than 18 or older than 45 years; known autoimmune disease; previous thyroid disease; HIV positivity; use of medications that affect immunity; chronic diseases, such as diabetes; serious mental illness, such as bipolar disorder, schizophrenia, untreated depression, or unresolved perinatal bereavement; body mass index (BMI) <20; history of hyperemesis; current multiple gestation; current pregnancy product of* in vitro* fertilization (IVF); fetal abnormalities; and being unable to participate in a six-month postpartum followup. All TPO positive (*n* = 63) women who were identified in pregnancy were invited into the postpartum phase of the study, while a sample of TPO negative women (*n* = 568) were selected by a random number generator to participate as the comparison group in the postpartum phase (*n* = 72). TPO status was determined by ELISA (ORGENTEC, Mainz, Germany) according to kit directions using standards and controls and done in duplicate. The coefficient of variation was always less than 5%. TPO antibody titer greater than 20 IUs was used as the cutoff value for positivity. TSH levels were also measured in all TPO positive women at the initial pregnancy blood draw. Women were not informed of their TPO status until the end of the study, or unless they experienced PPT and needed referral.

## 3. Materials and Procedure

Women recruited into this study received a home visit by a research nurse one week after giving birth and then monthly for postpartum months 1 through 6. At each postpartum home visit, participants received a targeted physical examination (thyroid gland palpation, blood pressure, heart rate, and weight). Detailed data were gathered on breastfeeding and smoking, alcohol consumption, and exercise. They also completed a thyroid disease symptom list. This list consisted of 19 items and participants rated occurrence of symptoms as not present (0), mild (1), moderate (2), severe (3), or very severe (4) (Table 1). The list was developed by the investigators based on a review of literature on symptoms of thyroid disease, a review of older clinical screening instruments which were deemed too difficult to use in the home setting [8], and consultation with an endocrinologist.

The Perceived Stress Scale (PSS) [9] was used to assess levels of self-reported stress and was completed at each postpartum visit. The 14-item version of the scale evaluates perceptions of stress using a Likert scale ranging from 0 (never) to 4 (very often). The internal consistency reliability has been reported to be .84 to .86 in young adults. Congruent and criterion validity for the scale has been demonstrated to be excellent, although predictive validity declines over time [9].

A venipuncture was performed and 15 mLs of blood were drawn into heparinized vacutainers. The blood was transported to the laboratory within 2 hours. Blood samples were centrifuged at 1200 g for 25 minutes at 4°C and plasma aliquoted into Eppendorf tubes and frozen at −80°C until later analysis in batches. TSH (ALPCO, Salem, N.H.) was always measured at each visit for the TPO positive women, regardless of symptoms, and assayed according to kit directions. TPO negative women with severity scores on the thyroid screening list indicating occurrence of hyper- or hypothyroid symptoms also had TSH levels measured. PPT was assessed as possible when a TSH level of less than 0.3 or more than 3.0 mIU/L occurred. This is the level chosen for screening of thyroid disease in adults [10] and during pregnancy [11]. Women meeting these criteria were referred to their health care providers for further diagnosis and treatment. They continued in the study after referral. The research nurse who made the postpartum home visits and the research participants were blinded to TPO status.

Statistical analysis was performed using SPSS v. 22. Data were analyzed descriptively and examined for normality and transformed if necessary. One way analysis of variance was computed on symptom subscale scores and PSS scores by group. Spearman correlations were used for correlations between TSH levels and signs and symptoms. A *P* value of <.05 was considered significant for these tests. Kruskal-Wallis analyses were performed on individual symptoms by group with a pairwise comparison test for post-hoc analyses. Bonferroni corrections were made for multiple comparisons with a *P* < .003 considered significant for these tests. Reliabilities (*α*) were calculated for the symptoms on the thyroid checklist hyperthyroid symptoms and hypothyroid symptoms subscales.

## 4. Results

TPO positivity was found in 63 of the 631 (10%) participants at the pregnancy measurement point. All but two of the TSH titers on these TPO positive women were in the euthyroid range when measured in the plasma samples collected in pregnancy (mean 1.46 ± .21; range 0.16–4.6 mIU/mL). Of the 63 TPO positive women, 46 agreed to be in the postpartum followup. The reasons for not participating in the postpartum follow-up study included moving to a different location, lack of interest in the study, and being lost to followup. The demographics of the TPO positive women who did not participate were not different than those who did participate. Of these 46 TPO positive participants, 26 (56.2%) developed PPT during the first six months of the postpartum. There were 4 TPO negative women who developed symptoms and signs of thyroid disease, including out of range TSH levels. These women were not included in the analysis as they could have developed another thyroid disease such as Graves' disease. The research was described to participants as a study of immunity and hormones. However, if a woman became PPT positive, she was informed of this result and referrals were made to her health care provider, so from that point on she did know her status.

**Figure 1:**
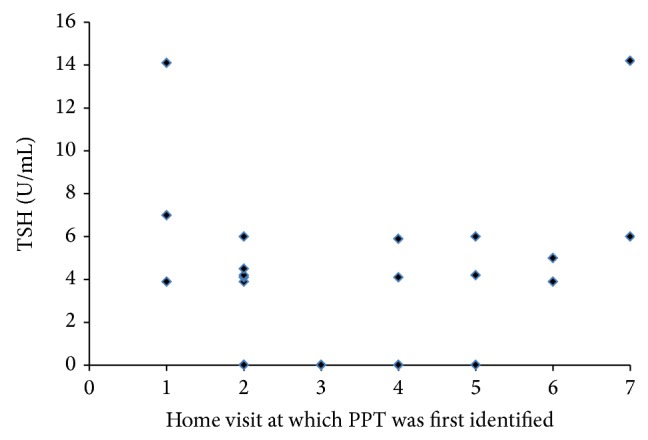
TSH levels in plasma at the time at which TPO positive women were first identified as PPT positive. Home visit 1 was at 1-week postpartum and the following time intervals were from month 1 through 6.

TSH levels were either below 0.3 mIU/mL or above 3.0 mIU/mL for a woman to be classified as having potential PPT. Thirteen had elevated TSH (hypothyroid) and thirteen had low TSH (hyperthyroid) at time of identification of possible PPT (Figure 1). While we attempted to assure followup with health care providers and potential further diagnostic testing for these women, we found that there is a common lack of knowledge about PPT by primary care providers, and few responded to our inquiries about follow-up diagnosis and treatment. In addition, some women had low incomes and no health care available for followup. For purposes of the study we classified TPO positive women as having likely developed PPT when a change (often dramatic) from normal TSH levels to levels outside the normal range occurred at one of the monthly visits and remained abnormal thereafter. No PPT positive woman, even those few who underwent medical referral and treatment, became euthyroid after being classified as PPT positive during the six-month course of the study. Only two PPT positive women were prescribed levothyronine and they continued in the study until the sixth month. Removing these women from the analysis had no effect on the results.

There were no differences in ethnicity by TPO status or PPT positivity status. Table 1 depicts sociodemographic variables by group. None of the differences were statistically significant.

The thyroid symptom list had both common hyperthyroid and hypothyroid symptoms which were grouped and analyzed as symptom severity subscales.  Table 2 shows symptoms categorized as being most associated with hypothyroidism or hyperthyroidism. The hyperthyroidism scale was comprised of 9 items (maximum severity score = 36) and the hypothyroidism scale had 10 items (maximum severity score = 40). The Cronbach's *α* for the hyperthyroid symptom subscale was .70 and for the hypothyroid subscale was .77. At time of first identification of PPT status, the mean score on the hypothyroid subscale was 8.5 ± 7.3 (SD) and on the hyperthyroid subscale was 5.4 ± 5.0 (SD). Comparisons of scores in the period before a woman became PPT positive showed a score of 6.75 ± 5.5 for the hypothyroid subscale and 6.87 ± 5.78 for the hyperthyroid scale, and these were not statistically significantly different from the values at identification of PPT positivity. However, the mean TSH of 1.52 ± .8 (SD) preceding the identification of PPT was significantly different (*t* = −3.0, *P* = .005) than the TSH at identification of presence of PPT (3.95 ± 3.96 (SD)).

**Figure 2:**
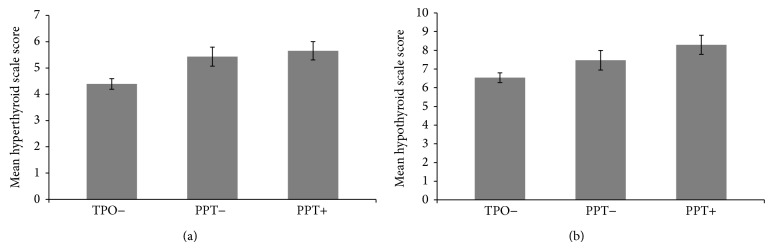
(a) Hypothyroid subscale scores by group. Error bars are standard errors of the mean. (b) Hyperthyroid subscale scores by group. Error bars are standard errors of the mean.

Figures 2(a) and 2(b) depict the means of the hypothyroid and hyperthyroid subscale scores across the postpartum for each group. A one-way between groups analysis of variance was conducted to explore the effect of group (TPO negative, TPO positive without PPT, and PPT positive) on the mean hypothyroid and hyperthyroid subscale scores. There was a statistically significant difference at the *P* = .003 level in hypothyroid subscale scores [*F* (2,742) = 5.8, *P* = .003]. Post-hoc comparisons using the Tukey HSD test indicated that the mean score for the hypothyroid subscale was significantly higher for the PPT positive group (8.29) compared to the TP0 negative group (6.53), but not significantly different than the TPO positive, PPT negative group (7.47). With regard to the hyperthyroid subscale, there was a statistically significant difference in scores at the *P* = .001 level [*F* (2,747) = 6.6, *P* = .001]. Post-hoc analysis found that there were significant differences in the hyperthyroid subscale scores comparing TPO negative women (4.39) with both TPO positive PPT negative (5.43) and PPT positive (5.65) groups.

All groups reported experiencing symptoms and there were no dramatic differences by group in the frequency of reporting any symptom experience (Table 3).  Table 4 depicts the frequency of women who reported that a symptom was either “severe” or “very severe.” Correcting for multiple comparisons by using a Bonferroni correction (*P* < .003), six symptom severities were significantly different by group (lack of energy, shaking hands, heat intolerance, cold intolerance, poor memory, and lack of concentration). All significant symptom severities were higher in both TPO positive and PPT positive groups compared to TPO negative women.

Twelve TPO positive women had palpable thyroid glands and 5 PPT positive women and two TPO positive, PPT negative, women had visible goiters. The five goiters in PPT positive women were associated with extremely low TSH levels in three women and high levels in two women.

Mean PSS scores were significantly different by group [*F* (2,755) = 11.1, *P* < .001]. Post-hoc analysis showed that PPT positive women had higher mean scores across the postpartum (22.5) compared to TPO negative women (19.3) (*P* < .001). However, the difference between PPT positive and TPO positive, PPT negative women (20.8) was not significant (*P* = .15).

In the sample as a whole, TSH levels were correlated with heart rate (*r* = .124, *P* < .05), systolic blood pressure (*r* = .164, *P* < .01), diastolic blood pressure (*r* = .22, *P* < .001), and BMI (*r* = .15, *P* < .02). The scores on hyper- versus hypothyroid subscales were not significantly correlated with TSH levels.

## 5. Discussion

Many postpartum women feel exhausted, depressed, and not able to concentrate. Even physical symptoms such as hair falling out were reported as occurring by a third of postpartum women who were TPO negative in the study. Certainly postpartum women are often dealing with many demands and stressors [12–14]. Differentiating the “normal” postpartum experience from the experience of women with thyroid disease cannot rely on symptom reports. However, severity of symptoms was clearly different for both groups of TPO positive women. The TPO positive women who did not develop thyroiditis were different in hypo- and hyperthyroid subscale scores compared to TPO negative women. They also had significant differences in particular symptom severities. The presence of measurable thyroid autoantibodies, whether or not PPT develops, indicates an autoimmune perturbation in the gland even in euthyroid women. We and others have reported that the presence of TPO autoantibodies alone may be associated with postpartum depression [15, 16]. Thyroid inflammation could have subtle effects on neurological function.

When PPT does develop, it is unlike other autoimmune diseases in that it is reversible, although many women who develop PPT go on to later chronic hypothyroidism. It is also an unusual disease in that as yet unknown postpartum physiological changes unmask the disease in susceptible women. The observation that perceived stress was significantly higher in the PPT group is noteworthy. The distress associated with the disease is likely additive with the usual stressors associated with the postpartum.

This study should raise the awareness of providers to the need to screen for TPO autoantibodies, since these women appear to be experiencing more postpartum distress and are at risk for development of PPT. Screening for TPO autoantibodies is currently not a clinical recommendation for all pregnant women and therefore many cases of PPT are missed. In our study, of the 26 who were identified with PPT, only two would have returned to their health care provider based on their perceptions of the severity of their symptoms. Even with referrals being made for all the PPT positive women in the study, only two were actually treated. Treatment could have included thyroid hormone replacement, antidepressants, and supportive care, but the usual approach was to watch and wait. The return to euthyroidism after PPT does not necessarily herald a healthy thyroid gland, as the risk for permanent thyroid disease as well as recurrence of PPT in subsequent pregnancies is very significant [17]. In addition, the quality of life for women with PPT as well as those who are TPO positive is likely to be impaired by severity of some of the symptoms we measured as well as by general perceived stress. Clearly there is a need for health care providers to be more aware of this condition, as it may appear after the usual 6-week postpartum visit and then may be present in the primary care setting.

## 6. Limitations

There are several important limitations to this study. The number of participants with PPT was small. The thyroid symptom list may have inadequately captured the symptoms of PPT, and the symptom and stress data were self-report. The designation of PPT status was based on a change from normal range TSH levels to out of range levels, without a full thyroid panel and clinical diagnosis. Women who became PPT positive were so informed and thus this may have influenced their responses in subsequent home visits.

Additional studies are needed to determine the most appropriate symptom screenings useful in diagnosing PPT. However, these data should prompt clinicians to investigate thyroid function when women report severe exhaustion, sleep disturbances, weight gain, and hair loss in the postpartum period. Attributing these symptoms to normal postpartum occurrences can cause unnecessary suffering for some new mothers.

## Figures and Tables

**Table 1:** Demographic characteristics by group.

	TPO negative	TPO positive	PPT positive
Income	9% < $4999	.6% < $4999	1.1% < $4999
	31% > $69,999	38% > $69,999	38% > $69,999
BMI	29.7	32.1	28.3
Breastfeeding	52%	70%	56%
Smoking	8%	1.6%	0%
Parity	1.03	1.00	1.00

**Table 2:** Thyroid symptom screening.

**Lack of energy**	0	1	2	3	4
Irritability	0	1	2	3	4
Nervousness	0	1	2	3	4
Sweating	0	1	2	3	4
Dry skin	0	1	2	3	4
Shaking hands	0	1	2	3	4
**Depression**	0	1	2	3	4
Heat intolerance	0	1	2	3	4
**Dry hair**	0	1	2	3	4
**Puffy face**	0	1	2	3	4
**Hair falling out**	0	1	2	3	4
**Weight loss**	0	1	2	3	4
**Constipation**	0	1	2	3	4
Aches and pains	0	1	2	3	4
Tingling or numbness	0	1	2	3	4
**Cold intolerance**	0	1	2	3	4
**Poor memory**	0	1	2	3	4
**Lack of concentration**	0	1	2	3	4
Palpitations	0	1	2	3	4

bold items comprise the hypothyroid subscale; non bold items comprise the hyperthyroid subscale.

0: no presence of symptom; 1: mild; 2: moderate; 3: severe; 4: very severe.

**Table 3:** Percent of women reporting any symptoms on thyroid symptom checklist by TPO and PPT status.

Symptom	TPO negative	TPO positive	TPO positive PPT positive
Lack of energy	68.4%	74.6%	78%
Irritability	49.6%	54.4%	61.9%
Nervousness	32.7%	37.9%	37.1%
Weight loss	44.5%	24.3%	48.7%
Sweating	31.5%	32%	38.6%
Shaking hands	11.6%	15.5%	10.7%
Palpitations	11.2%	11.7%	10.2%
Heat intolerance	31.9%	36.9%	41.6%
Dry hair	34.1%	34.9%	35.5%
Puffy face	13.2%	17.6%	14.2%
Dry skin	50.6%	55.3%	51.3%
Hair falling out	38.4%	54.4%	34%
Constipation	37.9%	37.9%	35.5%
Aches and pains	48.6%	44%	52.3%
Tingling or numbness	17.9%	13.6%	21.8%
Cold intolerance	32.8%	26.2%	21.2%
Poor memory	49.8%	48.5%	57.4%
Lack of concentration	44.4%	49.5%	57.9%
Depression	21.5%	29.1%	33.5%

**Table 4:** Comparisons of frequencies of “severe or vey severe” symptom reports on thyroid symptom checklist by group.

Symptom	TPO negative (a)	TPO positive PPT negative (b)	PPT positive (c)
Lack of energy	13.2%	18%	18% (∗∗a, c)
Irritability	9.5%	5.9%	11.4%
Nervousness	5%	5.2%	8.6%
Weight loss	3.9%	5.9%	8.0%
Sweating	4.4%	5.9%	9.2%
Shaking hands	1.8%	1.5%	6.1% (∗∗b, c)
Palpitations	0.08%	0%	1.2%
Heat Intolerance	5.5%	8.2%	8.6% (∗∗a, c)
Dry hair	5.1%	10.4%	12.3%
Puffy face	0.08%	0.08%	0.06%
Dry skin	11.9%	9.7%	12.8%
Hair falling out	13.5%	14.1%	14.7%
Constipation	7.1%	12.7%	13.5%
Aches and pains	10.4%	11.1%	11.7%
Tingling or numbness	3%	4.4%	2.5%
Cold intolerance	3.3%	2.2%	6.7% (∗∗b, c)
Poor memory	6.4%	10.4%	13.5% (∗∗a, c)
Lack of concentration	6.0%	4.4%	12.8% (∗∗b, c)
Depression	3.3%	8.3%	6.1%

∗∗indicate *P* < 0.003.
